# Cognitive remediation therapy (CRT-O) for young Indian adults living with overweight or obesity: a qualitative study of participant experience

**DOI:** 10.1007/s40519-026-01845-5

**Published:** 2026-03-27

**Authors:** Vandhana Susilkumar, Bhasi Sukumaran, Radha Kumar, Jayanthi Raman

**Affiliations:** 1 Saveetha Institute of Medical & Technical Sciences (SIMATS), Chennai, Tamil Nadu India; 2 SRM Medical College Hospital and Research Centre, SRM Institute of Science and Technology, Chengalpattu Dt., Kattankulathur, Tamil Nadu India; 3 School of Psychological Sciences, University of Newcastle, University Drive Callaghan, Newcastle, NSW 2308 Australia

**Keywords:** Obesity, Disordered eating, Cognitive remediation therapy (CRT-O), Qualitative analysis

## Abstract

**Background:**

The prevalence of obesity in India has reached epidemic levels with 40.3% living with overweight or obesity. Problematic eating behaviours have been implicated in the development and maintenance of overweight and obesity. Conventional cognitive-behavioural intervention for eating disorders has not shown favourable long-term outcomes in weight management. Recent research has indicated early evidence for using cognitive remediation therapy (CRT-O) for eating disorders including disordered eating behaviours in those living with overweight and obesity. We conducted a feasibility study through a randomized controlled trial (RCT) with 70 young Indian adults with obesity or overweight. We also obtained participant feedback post-intervention, to identify putative contributing factors to the study outcomes.

**Methodology:**

Fifteen young adult participants completed 28 sessions of CRT-O intervention program for their weight management. On trial completion, participants were randomly selected to provide their feedback and experience of their participation. A semi-structured qualitative interview was conducted. Transcripts were semantically coded and thematic analysis was done.

**Results:**

Thematic analysis yielded six themes: 1. gaining new insights into their eating habits; 2. self-reported changes in eating behaviours; 3. self-reported behavioural changes; 4. perceived physical benefits; 5. perceived psychological benefits; 6. perceptions about the CRT-program. The sub-themes that emerged indicated general acceptance of the CRT-O intervention and its potential translation in real life.

**Conclusions:**

This study showed improved metacognitive awareness on participants’ eating behaviours. Additionally, the findings indicated that participants developed an improved ability to resist several highly palatable but unhealthy foods. Participants also reported experiencing more self-control in their food grazing habits and reported that this led to a better ability to manage their weight.

## Introduction

Obesity is one of the most prevalent and modifiable non-communicable disorders and is a global health concern of the twenty-first century. According to the World Health Organization, around 2 billion adults are overweight, of whom 650 million are considered to be affected by obesity (BMI ≥ 30 kg/m^2^) which indicates that 39% of adults aged 18 or over live with overweight, and 16% live with obesity [1]. It has been predicted that in India the incidence of overweight among adults aged 20–69 years was estimated to double between 2010 and 2040 and the occurrence of obesity will triple during this time period. Specifically, the prevalence of overweight and obesity will be estimated to reach 30.5% and 9.5% among men, and 27.4% and 13.9% among women, respectively, by 2040 [2].

The aetiology and maintenance aspects of obesity are multifactorial and include various biological, psychological, and environmental factors. Current conventional interventions in the treatment of weight management in those living with overweight and obesity such as lifestyle behavioural interventions [3] and cognitive-behavioural interventions [4] have been shown to have poor longer-term outcomes.

Even when individuals lose clinically significant weight in the short-term, recidivism is high and this has been attributed to unhelpful weight management activities, such as lack of adherence to recommended physical activity and healthy eating behaviours [5]. Engaging individuals in weight management can be challenging as many feel ambivalence towards maintenance of weight due to anticipated relapses in their eating behaviour [6]. Given these challenges, it is important to explore innovative interventions. Current research in experimental and clinical psychology has implicated the role of cognitive aspects in obesity [8]. Recent studies have found that individuals with obesity demonstrate cognitive deficits, mainly in executive functions independent of age and associated comorbidities [7]. In particular, cognitive flexibility, planning, working memory and inhibitory control have been identified in obesity maintenance. Consequently, there is increasing research interest and early evidence in addressing cognitive deficits in those living with obesity [9].

Cognitive remediation therapy (CRT) emphasizes the improvement of cognitive functions, including working memory, and executive functions through a structured system of specific instructions and neurocognitive exercises [9, 26]. Cognitive Remediation Therapy for obesity (CRT-O) has recently been shown to improve executive functioning in those with eating disorders [10, 11, 27]. There is early evidence that CRT-O also improves binge eating in those with obesity [12, 13].

The current study aimed to investigate the feasibility of a CRT-O intervention in young Indian adults. We also aimed to obtain participant experiences after they completed a CRT-O intervention trial.

## Methods

### Study design

This study was an extension of a randomized controlled trial that evaluated the effectiveness of CRT-O among young adults with obesity. Participants were recruited from community settings including weight management clinic settings. The study was approved by the Institutional Ethics Committee of the Saveetha Medical College and Hospital (010/06/2021/IEC/SMCH).

The anthropometric markers, encompassing height and body weight, were measured in compliance with a standard operating protocol. Body mass index (BMI) was computed as per the WHO formula as weight (kg)/height^2^ (m^2^) and was categorized as overweight (BMI 25–29), obese (BMI 30–34). Participants with overweight (BMI 25–29) or obesity (BMI > 30) were included in the CRT-O study. Out of 35 participants in the intervention group, 15 participants were randomly selected by drawing lots to participate in the qualitative study, among them 4 in the obesity category and 11 in the overweight category. The inclusion criterion for the qualitative study was the completion of 28 days of the CRT-O intervention program. Semi-structured interviews took place following successful completion of the intervention. Baseline demographic information including age, gender, education and marital status was collected along with anthropometric information (height, weight). Mental health was assessed using the Depression, Anxiety, and Stress Scale-21 (DASS-21), while eating habits and behaviours were evaluated with the Grazing Questionnaire (GQ), Self-Regulation in Eating Behaviors Scale (SEREB), Eating Disorder Examination Questionnaire (EDE-Q), and the Three-Factor Eating Questionnaire (TFEQ). Cognitive domains were measured using the Detail and Flexibility Questionnaire (DFlex) and a battery of neuropsychological tests, including the Auditory Verbal Learning Test (AVLT), Stroop Color and Word Test (STROOP), Trail Making Tests A and B (TMT-A, TMT-B), and the Letter-Number Sequencing (LNS) task.

### Cognitive remediation therapy-obesity (CRT-O) intervention

The manualised CRT-O intervention program aimed to address cognitive deficits that have been shown to be associated with obesity. The current manual was adapted and modified based on a study by Raman et al. [16, 17]. CRT-O intervention contents include: 1. introduction, 2. goal-setting, 3. selective attention and switching task, 4. inhibition training, 5. decision-making, 6. planning, 7. problem solving. Additionally, the CRT-O manual also consisted of supplementary homework activities and tasks that were given to the participants at the end of each session. Given the close association between working memory and executive function [15] our modification included working memory tasks. The modified manual addressed difficulties related to cognitive flexibility, planning, working memory and included exercises related to impulsivity.

Each participant received Cognitive Remediation Therapy for Obesity (CRT-O) consisting of three individual sessions per week over a 4-week period, in addition to 16 days of structured home-based activities. The home-based component included approximately four to five game-based cognitive exercises designed to reinforce and generalize the cognitive strategies introduced during in-person sessions. Each in-person CRT-O session lasted approximately 45 min and was followed by a debriefing discussion, during which participants reflected on their experience with the tasks, the cognitive and behavioural strategies they employed, and explored ways to apply these skills in their daily routines and eating-related situations. Each activity in a session focused on a particular cognitive aspect, the degree to which participants faced difficulties with cognitive flexibility or set-shifting, and each of these was discussed during the in-person session.

The purpose of CRT-O sessions was to determine participants’ cognitive styles, challenge ineffective thought patterns, and assist them in exploring different ways of thinking. It also aimed to encourage metacognitive awareness, or thinking about thinking, through a range of exercises and reflective questions.

In each of the sessions, a variety of exercises were included like games, puzzles, paper and pencil exercises to improve metacognitive awareness and to challenge ineffective thinking processes. Following each exercise, questions that encouraged reflection on thought processes, discussed alternate approaches to improve thinking style, and linked to changes made in real-world situations were asked.

The researcher-therapist adopted a non-biased approach in recognising the participants’ cognitive strengths and shortcomings while also exploring whether the participants’ cognitive style interfered with their day-to-day functioning. The researcher-therapist stressed the significance of focussing on the pattern of thinking instead of the level of performance to avoid any performance-related anxiety. Overall, participants engaged in CRT-O activities for a total duration of 28 days, offering a comprehensive combination of therapist-guided sessions and self-directed cognitive exercises aimed at enhancing executive function, cognitive flexibility, and self-regulation of eating behaviours. 

### Data collection and analysis

Semi-structured interviews were conducted with each participant after the completion of the 28 days CRT-O. The time interval between the first and second administration of the test measures was between 10 and 14 days with an average of 12 days after the completion of the study intervention. Open-ended questions were included and participants were requested to provide feedback. Information regarding the participants’ CRT-O experiences, what they found helpful, the likeability of the tasks and also further suggestions for CRT-O improvements was collected.

Participants were advised that the purpose of the interview was to provide feedback of their CRT-O experiences. The participants were asked to write their feedback in a form identified with a serial number, without mentioning their names. The interviews lasted for an average of 30 min.

Participants were encouraged to write about treatment utility; whether and how the intervention was applicable to everyday life; whether they would recommend it to others; and the ways in which they felt the intervention could be improved. Participants were encouraged to be as honest as they could be, and it was made clear that a new intervention was being tested and that any suggestions they made could be used to make it better.

Transcripts were imported into Nvivo 20 for management and analysis using Atlas Ti software. Reflexive Thematic Analysis (RTA), as described by Braun and Clarke [22, 24] was used as the framework when analysing the data following their six-phased process. Themes and sub-themes were developed using the analysis. Coding was done at the semantic level initially, and at the later stages the codes were refined and themes and sub-themes were created.

## Results

Fifteen participants were included in the qualitative study after completion of 28 sessions of CRT and obtaining informed consent. The sociodemographic details of the participants are shown in Table 1.

**Table 1:** Sociodemographic data of the participants

Characteristics		*N* = 15		Mean	SD	Test value	*p*-value
		*N*	%				
Gender	Male	4	26.70	–	–	–	–
	Female	11	73.30				
Age	Age (years)	15	100	22.13	2.39	–	–
BMI (Pre)	Overweight	11	73.30	27.65	1.59	t (3.86)	^**^0.002
	Obesity	4	26.70	31.43	1.95		
BMI (Post)	Normal	3	20.00	24.07	1.08	F (14.31)	^***^0.001
	Overweight	11	73.30	27.65	1.51		
	Obesity	1	6.70	32.54	–		

Variables of significance (*p ≤ 0.05, **p ≤ 0.01, ***p ≤ 0.001)

Among the 15 participants, 4 were males and 11 were females with educational status being college-level. The mean age was 23.13 and all the participants were unmarried (Table 1).

Table 2 presents the pre and post-intervention scores obtained by the participants on the various psychological, cognitive, and behavioural measures. There was a statistically significant reduction in BMI following the intervention, indicating a measurable improvement in body composition. However significant differences were found between the pre and post scores on 10 measures, with post-intervention scores being significantly lower than the pre-intervention scores. Cognitive flexibility showed a significant improvement following the intervention, reflecting enhanced mental adaptability and executive control. Participants also demonstrated a significant reduction in uncontrolled eating, indicating better self-regulation of eating behaviour. Across all learning and memory domains, there were significant post-intervention gains, suggesting enhanced cognitive performance and information retention. Additionally, executive function, particularly task-switching and mental flexibility as assessed by the TMT-B, improved markedly. Finally, there was a highly significant enhancement in working memory capacity, reflecting strengthened cognitive processing and short-term information management abilities.

**Table 2:** Pre- and post-intervention changes in psychological, cognitive, and behavioural measures

Variable	Category	Pre		Post		Paired *t*-test	*P*-value
		Mean	SD	Mean	SD		
BMI	BMI	28.66	2.37	27.26	2.47	4.12	0.001
DASS	Depression	17.33	12.23	9.73	9.56	2.06	NS
	Anxiety	16.67	10.55	16.00	7.71	0.18	NS
	Stress	22.53	11.75	16.53	10.86	1.36	NS
GQ	Grazing behaviour	5.73	2.55	3.40	2.16	1.41	NS
	Controllability	5.20	2.70	4.13	2.92	1.68	NS
	Total	10.93	4.83	7.53	4.64	1.69	NS
DFLEX	Cognitive flexibility	44.07	12.44	38.20	9.40	2.61	0.02
	Attention	38.60	10.70	35.93	5.40	0.97	NS
SEREB	SEREB total	82.40	16.77	62.33	10.46	3.39	0.004
EDEQ	Total	18.87	6.36	14.67	6.90	1.42	0.18
TFEQ	Cognitive restraint	46.94	11.30	42.22	13.26	1.11	NS
	Uncontrolled eating	29.07	11.78	18.89	7.59	2.44	0.03
	Emotional eating	31.11	22.38	27.22	24.29	0.41	NS
AVLT	Learning score	47.73	7.83	57.27	7.82	3.56	0.003
	Memory score	11.27	2.31	12.87	1.41	2.37	0.03
	Retention score	11.80	2.54	13.67	1.95	3.44	0.004
FAS	FAS score	37.40	10.58	39.73	7.37	0.83	NS
STROOP	Interference score	32.80	7.36	37.60	14.77	1.07	NS
TMT A	Time	38.33	12.83	30.93	12.04	1.62	NS
	Error	1.53	1.92	1.53	1.93	–	–
TMT B	Time	73.73	18.20	54.93	7.55	3.84	0.002
	Error	3.20	2.15	0.47	0.64	4.64	0.000
LNS	Retention score	19.00	2.90	26.20	2.08	9.36	0.000

*DASS-21* depression, anxiety stress scale-21, *GQ*-grazing questionnaire, *DFlex* detail and flexibility questionnaire, *SEREB* self-regulation in eating behaviours scale, *EDEQ* eating disorder examination questionnaire, *TFEQ* three-factor-eating-questionnaire, *AVLT* auditory-verbal learning test, *STROOP* Stroop Color and Word Test, *TMT A* trail making task A, *TMT B* trail making task B, *LNS* letter-number sequencing

### Thematic analysis

**Fig. 1:**
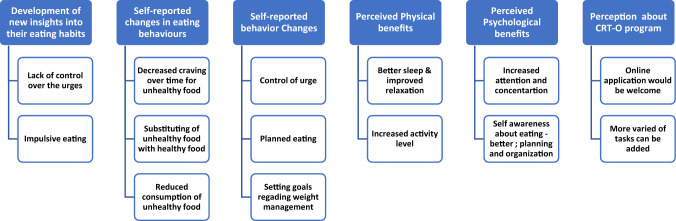
The structure of the themes and sub-themes

Six main themes and 14 sub-themes were found through the thematic analyses. The structure of the themes and sub-themes is presented in Fig. 1.

The six main themes were 1. development of new insights into their eating habits; 2. self-reported changes in eating behaviours; 3. self-reported behaviour changes; 4. perceived physical benefits; 5. perceived psychological benefits; 6. perceptions about the CRT-O Program.

1. Development of new insights into their eating habits

Most of the subjects reported that they started to think about their eating patterns and also make plans about what they eat *“I am able to track my macro nutrients and calories.”* They also mentioned impulsive decision-making with regard to what to eat, which eventually they regretted.

Subjects also reported that they had difficulty in resisting the urge to eat food and easily gave in to these temptations. Awareness of these patterns helped them to bring about changes in their eating behavior—*“self-control over food and binge eating has increased”—“helped me to stop, think, and act”.*

2. Self-reported changes in eating behaviours

Common changes reported were that subjects started to track and plan their dietary intake, and the quantity of food purchased as well as the portion size of the food they ate. They also mentioned tracking the calorie count of their food purchases. Subjects also started to plan healthy meals for the day by substituting “junk” food with healthier alternatives—“*able to switch from junk food to heathier alternatives”—“nowadays I am able to substitute it with dates or banana smoothie”.*

3. Self-reported behaviour changes

Improved decision-making and planning contributed to a variety of behaviour changes as mentioned by subjects. They reported an improved ability to say No to unhealthy foods, which enabled them to make healthier food choices. Some subjects also reported that “*my consumption gradually decreased”*. Improved planning and execution of the plan were also commented on: “*Planning was executed effectively without any procrastination”* “able *to switch to alternatives during emotional situation”.* Improved awareness about eating habits and being conscious about health conditions were the positive outcomes outlined.

4. Perceived physical benefits

Some of the physical benefits reported included being calmer and more relaxed, having better sleep and increased activity levels. A reduction in appetite was reported as well as an improved ability to manage cravings and switch to healthier alternatives which led to better control over food intake as well as weight management. The majority of the participants reported improved subjective physical wellbeing.

*“Sessions gave me an insight to be more conscious and be aware of my activities. Therefore, I have set goals to bring my weight down” “I get a good amount of sleep” “The whole process was mind relaxing” “whole process seemed to be enjoyable and nice to me and helped me to be active around even when I feel like stressed”. “Appetite has decreased substantially from 2 rotis to 1 roti”*

5. Perceived psychological benefits

Improvements in self-awareness and concentration levels were commonly listed by the participants. Participants mentioned that they became more conscious about their habitual patterns in terms of eating and were able to bring about the desired changes in food intake. This was enabled by better planning and organization, the ability to control temptations as well as improved flexibility, enabling the shift to making alternative choices. Subjective memory benefits were also mentioned.

“My concentration level has increased by doing these tasks. I can focus in particular without distraction.” “Able to focus consciously on minute things which I wasn’t aware earlier.” “Helped me to plan, organize and also control on my craving for food”.

“I’ve gained the habit of thinking before acting,” My memory is been improved and able to focus more on work”. “I have developed patience and the tasks which seems to be difficult for me prior now seems to be easier”

6. Perceptions about the CRT-O Program

Paper pencil tasks and activities were enjoyed by the participants who found them interesting and also applicable to day-to-day life. They were able to plan before doing a task which was reflected in subtle changes in eating habits. Better knowledge in controlling focus and improved planning skills were commonly mentioned.

“The work sheets were very interesting and felt like a video game” “I liked the nondominant hand activities such became easier day by day.” “Activities and the process were enjoyable “

*“I began to realise the desired changes being implemented in my day-to-day life.”*

Table 3 represents the six thematic categories matched with representative quotations to enhance understanding of the diversity of participants’ perspectives linked to individual participant codes. The inclusion of direct participants’ feedback enhances the richness and authenticity of the findings, providing deeper insight into how individuals perceived and integrated cognitive remediation strategies into their daily routines.

**Table 3:** Thematic categories matched with representative quotations

Main theme	Representative quotations	Participant
1. Development of new insights into eating habits	“self-control over food and binge eating has increased”—“helped me to stop, think, and act”	EO
	“CRT sessions gave me an insight to be more conscious and aware of my activities.”	MS
	“I was able to consciously focus on minute things which I wasn’t aware of earlier.”	AR
	“I think a lot before eating something and am very conscious about my health condition.”	RA
	“I am able to track my macronutrients and calories.”	NP
2. Self-reported changes in eating behaviours	“I am able to switch from junk food to healthier alternatives.”	VS
	“Nowadays I am able to substitute sweets with dates or banana smoothies.”	JA
	“My appetite has decreased substantially from two rotis to one roti.”	NI
3. Self-reported behavioural changes	“I am able to say ‘no’ to almost every junk food.”	SW
	“Planning was executed effectively without any procrastination.”	MA
	“My consumption gradually decreased.”	SB
4. Perceived physical benefits	“I get a good amount of sleep and now started eating breakfast rather than skipping it.”	SW
	“The whole process was mind-relaxing and helped me stay active even when I felt stressed.”	VS
	“Reduced 2 kg weight, feeling more active than before.”	MS
	“My appetite has come down.”	RA
5. Perceived psychological benefits	“My concentration level has increased by doing these tasks. I can focus in particular without distraction.”	SV
	“I have developed patience, and the tasks that seemed difficult earlier now seem easier.”	NI
	“My memory has improved, and I am able to focus more on work.”	EO
	“I’ve gained the habit of thinking before acting.”	LA
6. Perceptions about the CRT-O Program	“The worksheets were very interesting and felt like a video game.”	JA
	“The activities and the process were enjoyable and applicable to day-to-day life.”	VS
	“I liked the non-dominant hand activities, which became easier day by day.”	RA
	“It was a wonderful experience; it helped me a lot in planning and decision-making.”	JI

Table 4 shows there were no participants in the normal-weight group at baseline; however, they were categorized as overweight (*F *= 9, *M* = 2) or obese (*F* = 2, *M* = 2). The overweight group’s mean BMI remained unchanged after the intervention, whereas the obese group’s BMI did marginally increase. Notably, after the intervention, three subjects transitioned from the obesity group to the normal-weight category.

**Table 4:** Summary of participants with overweight and obesity

Characteristics		Pre-intervention	Post-intervention	Change in BMI
Overweight	Gender	*F* = 9 (N) *M* = 2 (N)	*F* = 9 (N) *M* = 2 (N)	Nil
	BMI	27.65 ± 1.51	27.65 ± 1.51	
Obesity	Gender	*F* = 2 (N) *M* = 2 (N)	*F* = 2 (N) *M* = 1 (N)	Nil
	BMI	31.43 ± 1.95	32.54 ± 0	
Normal weight	Gender	No normal-weight participants during enrolment	*F* = 2 (N) *M* = 1 (N)	BMI of 3 participants shifted from obese to normal-weight category
	BMI		24.07 ± 1.08	

## Discussion

Emerging research has indicated the potential application of CRT for weight management, particularly as an adjunct therapy along with other behavioural interventions. The qualitative experience of CRT intervention has been well documented in anorexia nervosa [14]. The current study was carried out to evaluate the self-reported experiences of CRT-O intervention among young, Indian adults with overweight and obesity using thematic analyses [18, 25]. Several participants reported that CRT-O was enjoyable as it was completely game-based activities and tasks, in which the focus of discussion was not on food or eating behaviours. These findings are in line with results reported by previous studies. [19, 21]. Two previous studies had shown that CRT was helpful in reducing rigid ways of thinking in terms of eating and that participants were able to come up with a plan of alternate ways of eating a healthy diet. [20, 21]. In the current study, participants reported similar effects; these included being more aware of their impulsive patterns of eating, an improved ability to progressively harness the strong urges that they previously found difficult to control, as CRT-O sessions progressed, and these facilitated changes in their eating choices. Rigidity in thinking prior to CRT and development of flexibility in thinking commonly emerged as major findings in the current study. Participants also reported that they became aware of their patterns of eating and this awareness helped them to shift to healthier choices. Similar findings have been reported in previous qualitative studies on CRT with eating disorders and obesity. [20, 21]

Most participants reported the sessions were enjoyable and reported feeling refreshed after performing the CRT-O tasks. Some participants stated that online availability of CRT-O would be useful and more accessible. Similar feedback has also been highlighted in a study conducted on eating disorders [23].

## Strengths, limitations and future research directions

To our knowledge, this is the first study in India to explore participants’ experience on a modified CRT-O Program. This is a major strength of the current study as it provides important insights about the adaptability of the intervention in the Indian context. Participants’ feedback regarding the intervention was positive. In particular, the pragmatic application of the skills received positive feedback.

Our study findings are in line with previous studies of CRT and add important empirical evidence to develop new intervention pathways in the complex field of eating disorders. The study showed that participants acquired important metacognitive skills through the CRT-O exercises, and were able to apply this to their day-to-day activities. Another strength of this study was that recruitment was from the general community. An important limitation was that we did not exclude participants who were engaged in weight management programmes and due to the small sample size, we did not stratify the results accordingly. Future studies should aim to gather larger data in this context. Another recommendation for future studies is also to implement the CRT-O program in those with Class II and Class III obesity. The outcomes of treatment in a larger group incorporating wider age ranges, as well as over time, still need to be evaluated. The current study findings are limited to young Indian adults with overweight and obesity between the age range of 18–25 years and hence could not be generalized to other eating disorders. On a cultural note, while most metropolitan cities in India offer an acceptable representation of various communities, significant cultural differences may still influence behaviours. Future research should also consider cultural dimensions of food and eating practices to better understand these variations in study findings.

## Data Availability

The datasets generated during the current study are available from the corresponding author.
